# Corrigendum to Incoherence in the Brain Death Guideline Regarding Brain Blood Flow Testing: Lessons from the Much-Publicized Case of Zack Dunlap

**DOI:** 10.1177/00243639251328388

**Published:** 2025-04-13

Nguyen, Doyen, and Christine M. Zaine. 2025. Incoherence in the brain death guideline regarding brain blood flow testing: Lessons from the much-publicized case of Zack Dunlap. *The Linacre Quarterly*. doi:10.1177/00243639251317690.

For the above-mentioned article, the acknowledgment section has been updated. The correct acknowledgment reads as follows:

